# *CoreCruncher*: Fast and Robust Construction of Core Genomes in Large Prokaryotic Data Sets

**DOI:** 10.1093/molbev/msaa224

**Published:** 2020-09-04

**Authors:** Connor D Harris, Ellis L Torrance, Kasie Raymann, Louis-Marie Bobay

**Affiliations:** Department of Biology, University of North Carolina Greensboro, Greensboro, NC

**Keywords:** core genome, prokaryotes, orthology

## Abstract

The core genome represents the set of genes shared by all, or nearly all, strains of a given population or species of prokaryotes. Inferring the core genome is integral to many genomic analyses, however, most methods rely on the comparison of all the pairs of genomes; a step that is becoming increasingly difficult given the massive accumulation of genomic data. Here, we present *CoreCruncher*; a program that robustly and rapidly constructs core genomes across hundreds or thousands of genomes. *CoreCruncher* does not compute all pairwise genome comparisons and uses a heuristic based on the distributions of identity scores to classify sequences as orthologs or paralogs/xenologs. Although it is much faster than current methods, our results indicate that our approach is more conservative than other tools and less sensitive to the presence of paralogs and xenologs. *CoreCruncher* is freely available from: https://github.com/lbobay/CoreCruncher. *CoreCruncher* is written in Python 3.7 and can also run on Python 2.7 without modification. It requires the python library Numpy and either *Usearch* or *Blast*. Certain options require the programs *muscle* or *mafft*.

## Introduction

The core genome is defined as the set of genes that are ubiquitous—or nearly ubiquitous—to a set of genomes (Medini et al. 2005; Vernikos et al. 2015). Analysis of prokaryotic genomes often requires identifying the core genome of a species or a population to reconstruct strain phylogeny and to infer various metrics (Bobay and Ochman 2018; Maistrenko et al. 2020). Multiple tools have been built to generate core genomes and these approaches usually require the identification of orthologous genes by identifying best-bidirectional hits (BBH)through the comparison of each pair of genomes (Li et al. 2003; Kristensen et al. 2011; Miele et al. 2011; Contreras-Moreira and Vinuesa 2013; Page et al. 2015). Due to the massive accumulation of complete bacterial genomes, it has become computationally challenging—if even possible—to perform all pairwise comparisons when data sets include hundreds to thousands of genomes for a given species (Kristensen et al. 2011). As a result, alternative approaches are needed to efficiently process large data sets (Page et al. 2015). Several heuristics have been developed to address these challenges; however, very few tools have been designed to construct core genomes specifically. Instead, these tools usually aim to define the entire pan-genome (i.e., the entire set of genes in a given set of genomes) (Page et al. 2015). Because they aim to build the entire set of homologs; these methods are typically much slower.

One central challenge in defining the core genome is the correct inference of orthologous versus paralogous and xenologous genes (Chen et al. 2007; Altenhoff and Dessimoz 2009, 2012). Prokaryotes frequently undergo duplication, and more predominantly, horizontal gene transfer (HGT) events which may introduce paralogs and xenologs, respectively (Treangen and Rocha 2011). Paralogous and xenologous sequences can subsequently be lost by deletions or due to assembly issues and, as a result, even single copy genes may not represent true orthologs. Accurate distinction between orthologs and paralogs/xenologs is needed to build core genomes composed solely of orthologous genes. Traditionally, two main categories of methods are used to identify orthologs: graph- and tree-based approaches (Altenhoff and Dessimoz 2012; Sonnhammer et al. 2014). Core genomes are typically built for genomes of the same prokaryotic species—of which conspecific strains frequently engage in homologous recombination (Bobay and Ochman 2017). Due to the frequency of recombination, tree-based approaches offer little power to distinguish orthologs and paralogs/xenologs making this method much better suited to define orthologs across different species or lineages (Bobay and Ochman 2017). Alternatively, many graph-based methods exist, though most have been implemented for the general purpose of identifying orthologs in diverse contexts and often aim at identifying orthogroups that may include paralogs and xenologs (Tatusov et al. 1997; Remm et al. 2001; Li et al. 2003; Jothi et al. 2006; Kriventseva et al. 2007; Roth et al. 2008; Huerta-Cepas et al. 2016; Lafond et al. 2018; Cosentino and Iwasaki 2019). The identification of broader orthogroups is often desirable for analyses aimed at reconstructing the evolution of a gene family or when building the pan-genome of a species. Because core genomes are typically used for phylogenomic analyses and for the inference of population parameters, core genes are typically defined as “true” orthologs (i.e., without paralogs and xenologs).

Here we have developed *CoreCruncher*; a heuristic that quickly and robustly infers core genomes across large data sets of prokaryotic genomes. The key innovation of our algorithm relies on the implementation of a flexible test to distinguish paralogs and xenologs from orthologs by using the distributions of identity scores of homologous sequences to classify sequences as true orthologs or paralogs/xenologs. *CoreCruncher* is fast, has many customizable parameters, and can build the core genome of large data sets comprising thousands of genomes. In addition, the *CoreCruncher* algorithm may be used to identify sets of shared orthologs across divergent species; which expands its role beyond core genome assembly alone.

### New Approaches

Due to the accumulation of sequencing data, it has become common place to analyze hundreds to thousands of complete genome sequences during the study of a single prokaryotic species (Parks et al. 2018). Most algorithms implemented to define orthologous genes first rely on pairwise genome comparisons; a task that is becoming increasingly difficult to complete as data sets grow in size (Kristensen et al. 2011). To circumvent this issue, we have developed an approach that does not conduct all pairwise genome comparisons and instead, robustly identifies core genomes based on our “double outliers” approach to distinguish true orthologs from paralogs and xenologs using the distributions of identity scores.

In prokaryotes, the vast majority of new gene copies are gained by HGT (i.e., xenologs) (Treangen and Rocha 2011) which leads to the introduction of sequences that are expected to present atypical features relative to true orthologs (i.e., typically more divergent sequences) and additionally, are often found at different genomic locations (Gao and Miller 2020). Note that complex patterns of gene gains and loss can make paralogs and xenologs virtually indistinguishable. To robustly exclude paralogs and xenologs, our method first identifies homologous sequences by comparing each genome against a pivot genome. Putative orthologs are first built by assigning the best hit of each genome that matched the same gene of the pivot genome. This step yields putative orthologous gene families without within-paralogs/xenologs (i.e., paralogs or xenologs present in the same genome). When present, other hits are stored in memory and classified as within-paralogs/xenologs.

**Fig. 1.**
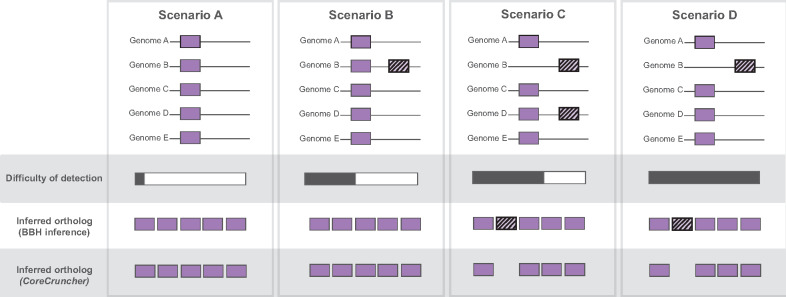
Impact of paralogs and xenologs on the inference of gene orthology. Scenario (*A*): No paralogs/xenologs. The orthologous gene is present in all the genomes; no paralogs or xenologs are present. Scenario (*B*): Within-paralogs/xenologs. The orthologous gene is present in all the genomes; one or more within-paralog/xenolog sequences are present. Scenario (*C*): Partially hidden paralogs/xenologs. The orthologous gene is missing in some genomes; some genomes are missing the orthologous sequence but contain a paralogous or a xenologous sequence (hidden paralog/xenolog); other genomes contain both the orthologous sequence and the paralogous or xenologous sequence (within-paralog/xenolog). Scenario (*D*): Completely hidden paralogs/xenologs. Some genomes are missing the orthologous sequence but contain a paralogous or a xenologous sequence (hidden paralog/xenolog); no within-paralogs/xenologs are present in other genomes. Plain boxes represent orthologous sequences; striped boxes represent paralogous or xenologous sequences. Scenarios *A* and *B* are expected to yield straightforward core gene predictions by BBH-based methods and *CoreCruncher*. Scenarios *C* and *D* will likely lead to the inclusion of paralogous and xenologous sequences in the core genome constructed with BBH-based approaches.

The first step of our procedure consists of identifying partially hidden paralogs/xenologs (fig. 1*C*). Partially hidden paralogs/xenologs occur when 1) one or more genomes lack the orthologous sequence of the gene but contain a paralog or a xenolog and 2) one or more genomes contain both copies of the orthologous sequence and the paralogous or xenologous sequence. Because at least one or more within-paralogs/xenologs are present in some of the genomes, our procedure uses the distribution of the identify scores of the within-paralogs/xenologs to identify partially hidden paralogs/xenologs present in other genomes (see Materials and Methods section).

**Fig. 2.**
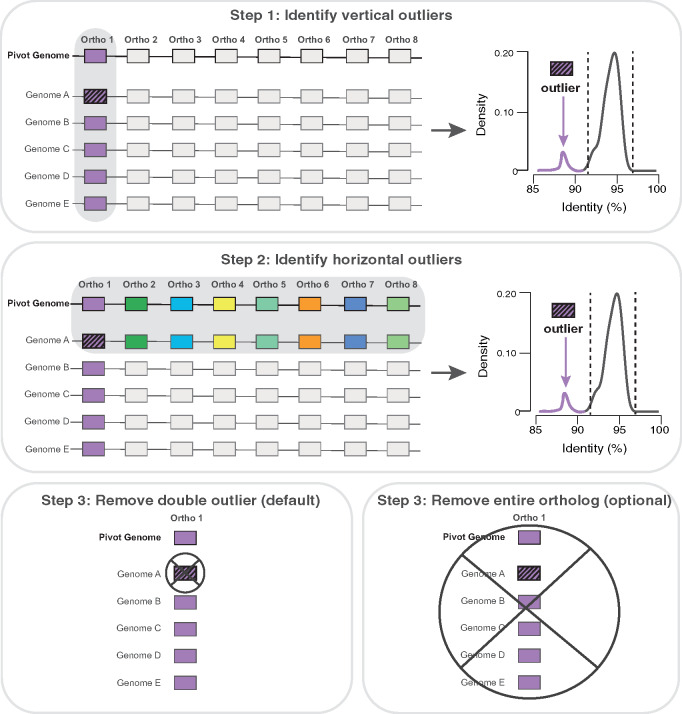
Identification of paralogs and xenologs with the double outlier test. *CoreCruncher* systematically tests for the presence of hidden paralogs/xenologs in each putative core gene. A sequence is inferred as paralogous/xenologous if it is identified as a vertical outlier and a horizontal outlier. Step 1. Vertical outliers: *CoreCruncher* builds distribution 1 for the putative core gene: the distribution of the identity scores of the best hit of each genome against the gene of the pivot genome. A sequence is considered as an outlier using Tukey’s fences: if its identity threshold is below Q1 − 1.5(Q3–Q1) or above Q3 + 1.5(Q3–Q1), with Q1 and Q3 the values of the first and third quartiles, respectively. Step 2. Horizontal outliers: sequences identified as outliers in step 1 are tested for the presence of horizontal outliers. *CoreCruncher* builds distribution 2 for the genome with a putative paralog/xenolog (i.e., an outlier was detected in step 1). The distribution is built by including all the putative orthologs of the genome with the putative paralog/xenolog against the pivot genome. The putative paralog/xenolog is considered a true paralog/xenolog if its identity score is also an outlier in distribution 2 using Tukey’s fences (see above). The paralog(s)/xenolog(s) inferred by the double outlier procedure is (are) then removed from the putative core gene. The putative core gene will be considered part of the core genome if present above the set frequency threshold used to define core genes (90% of genomes by default). When run with the stringent option, *CoreCruncher* will exclude any putative core gene with a paralog/xenolog identified with the double outlier test.

The second step of our procedure consists of identifying completely hidden paralogs/xenologs (fig. 1*D*). Completely hidden paralogs/xenologs occur when 1) one or more genomes lack the orthologous sequence of the gene but contain a paralog or a xenolog and 2) none of the genomes contain both copies of the orthologous and the paralogous/xenologous sequence. The presence of hidden paralogs/xenologs is detected with our “double outlier” procedure (fig. 2). Briefly, the distribution of identity scores (distribution 1) of each orthologous gene family is used to detect sequences that present significant outliers. These outliers likely represent paralogs or xenologs, and it is also possible that true orthologs present more divergent sequences because some strains are more divergent than others. To account for the differences in strain divergence across the data set, *CoreCruncher* also builds the distribution of identity scores for each putative ortholog for each genome relative to the pivot genome. This distribution (distribution 2) is used to estimate the overall divergence of each genome relative to the pivot genome. For each orthologous gene family, a sequence is identified as a completely hidden paralog/xenolog only if it is inferred as a double outlier based on distributions 1 and 2 (see Materials and Methods section). Paralogous and xenologous sequences identified by our approach are then excluded from each orthologous gene family, which is considered as core gene when found in high frequency across genomes (90% of the genomes by default). When run with the stringent option, the entire orthologous gene family is excluded from the core genome when a hidden paralog/xenolog is identified.

## Results

We tested *CoreCruncher* on a data set of 484 genomes of *Serratia marcescens* downloaded from *RefSeq* on December 2019 (supplementary table S1, Supplementary Material online). Using protein sequences and *Usearch*, we built the core genome of the same data set using 12 different sets of parameters: the core genome was built with a sequence identity threshold of 70%, 90%, and 95%. For each sequence threshold, orthologous genes were considered part of the core genome if found in 90%, 95%, 99%, or 100% of the genomes. In parallel we built the core genome of the same data set with *Roary* v3.13.0 (Page et al. 2015) using the same set of parameters. *Roary* is a state-of-the-art program that simultaneously builds the pan-genome and core genome of prokaryotic species. We chose *Roary*, for comparison with *CoreCruncher*, as it can directly infer a core genome and it uses pairwise genome comparisons (BBH) to define orthologs; a methodology that is used by most programs to define orthologs (Kristensen et al. 2011). *Roary*, however, conducts a preclustering step that allows it to be substantially faster than many other existing programs (Page et al. 2015).

**Fig. 3.**
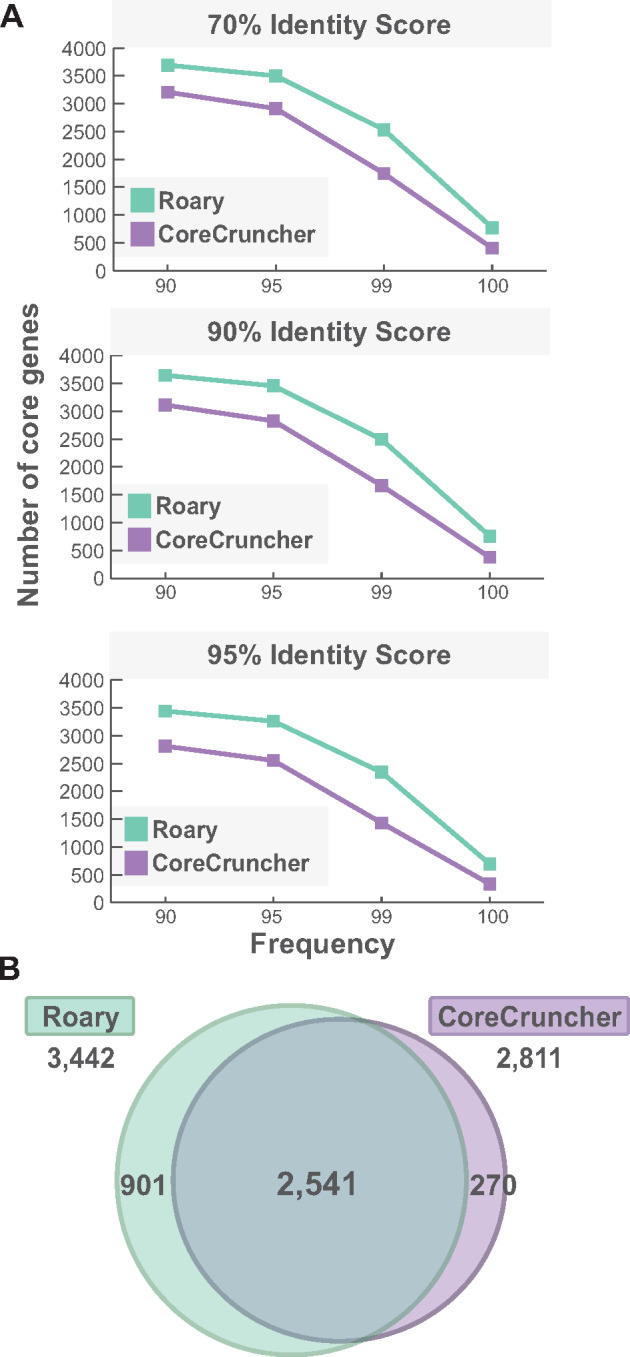
Comparison of the core genomes built by *CoreCruncher* and *Roary* across parameters. (*A*) Number of core genes inferred by *CoreCruncher* and *Roary*, respectively. Core genomes were built using a minimum sequence identity threshold of 70% (top), 90% (middle), and 95% (bottom). In each case, an orthologous gene family was considered as part of the core genome when present in at least 90%, 95%, 99%, or 100% of the genomes (*x*-axis). *CoreCruncher* was run using the nonstringent option and other parameters were set as default. (*B*) Comparison of the core genomes built by *CoreCruncher* and *Roary* using a minimum sequence identity threshold of 95% and a minimum genome frequency of 90%. Numbers indicate the number of core genes shared by both programs and specific to each program. Data for other parameters are presented supplementary table S2, Supplementary Material online.

We found that *CoreCruncher* systematically inferred a slightly smaller core genome than *Roary* for the same set of parameters (fig. 3). This difference can be ascribed to the fact that *CoreCruncher* uses more stringent parameters to build the core genome: 1) two sequences must present similar (≥80%) length to be inferred as homologs and 2) *CoreCruncher* can distinguish paralogous and xenologous genes from orthologous genes using the “double outlier” test. Indeed, *Roary*, like other related methods, has not been designed to detect hidden paralogs/xenologs. On average, the genes inferred as core by *Roary* and *CoreCruncher* were highly consistent (93.2% overlap on average, nonstringent option, and 94.9% overlap on average, stringent option, fig. 3, supplementary table S2 and fig. S1, Supplementary Material online).

We used the data set presented in figure 3*B* (95% sequence identity and 90% frequency) to analyze in more detail the orthologs that were inconsistently classified as either being part or not part of the core genome by the two programs. Overall, the majority of the core genes inferred by *CoreCruncher* that did not overlap with *Roary*’s core genome tended to correspond to two distinct orthologs defined by *Roary* (62.4% of the time). Interestingly, we found that the 270 core genes that were identified as core by *CoreCruncher*, and noncore by *Roary*, frequently contained paralogs or xenologs. By analyzing the *Usearch* output files, we found that 71% of these *CoreCruncher*-specific core genes contained at least one paralog/xenolog which is presumably why these gene families were not considered as core by *Roary*. However, *CoreCruncher* was able to sort out the orthologs from the paralogs/xenologs and still consider these genes as core (note that the paralogs/xenologs were not included in the core genome built by *CoreCruncher*). In contrast, only 44% of the genes inferred as core by both programs (shared core) presented one or more paralogs and/or xenologs (those were successfully excluded from the core by both programs). This suggests that the presence of paralogs and xenologs likely explains a large part of the discrepancies between the two programs. We further verified whether these *CoreCruncher*-specific core genes were true orthologs. We reasoned that if these core genes frequently included within-paralogs/xenologs or hidden paralogs/xenologs, many of them would present a wider distribution of identity scores. Therefore, we compared the core genes inferred by both programs to the 270 core genes inferred only by *CoreCruncher*. We aligned the sequences with *muscle* and computed the pairwise identify score for each core gene. We found no significant increase in the distribution of identity scores of the *CoreCruncher*-specific core genes relative to the core genes inferred by both programs; Wilcoxon tests on the distributions of 1) minimal values, 2) the standard variation, 3) the mean, and 4) the median values of identity scores (supplementary fig. S2, Supplementary Material online). We observed a slight but significant increase in the sequence length of the *CoreCruncher*-specific core genes (*P *<* *0.001, Wilcoxon test, supplementary fig. S2, Supplementary Material online), although we do not expect this difference to substantially affect the inference of the two programs. Overall, this analysis indicates that BBH-based programs like *Roary* are more likely to be affected by the presence of paralogs and xenologs and that the core genes inferred by *CoreCruncher*, and not by *Roary*, do not show evidence for the presence of paralogous or xenologous sequences.

The same data set was used to analyze the genes that were predicted as core by *Roary* and as noncore by *CoreCruncher* (901 genes, fig. 3). We found that a large portion of these genes (38%) were excluded from the core genome by *CoreCruncher* because they varied in length (by default, *CoreCruncher* imposes that sequences cannot differ by more than 20% in length). Very few of these genes (<1%) were excluded because the sequence was missing in the pivot genome used by *CoreCruncher*. Finally, 8% of these genes were excluded from the core genome of *CoreCruncher* because they contained paralogs or xenologs based on the “double outlier” test.

To further analyze the reasons for gene exclusion by *CoreCruncher* as compared with the same analysis done by *Roary*, genes were aligned with *muscle* and pairwise identity scores were computed for each of the 901 *Roary*-specific core genes. We found that most of these genes presented a much higher range of sequence identities relative to the 2,541 core genes inferred by both *Roary* and *CoreCruncher* (supplementary fig. S2, Supplementary Material online). Indeed, the minimal identity score, the average identity score, and the median identity score were significantly lower when compared with the core genes inferred by both tools (*P *<* *0.0001, Wilcoxon test, supplementary fig. S2, Supplementary Material online)—whereas the standard deviation of these identity scores were significantly higher (*P *<* *0.0001, Wilcoxon test, supplementary fig. S2, Supplementary Material online). Surprisingly, some of these *Roary*-specific core genes presented as low as 50% protein identity (supplementary fig. S2, Supplementary Material online). *Roary*’s inference of highly divergent sequences as part of the same core gene—despite using a threshold of 95% sequence identity—can be ascribed to the BBH procedure. The BBH approach infers pairs of sequences as orthologs based on a sequence identity threshold (95% in this analysis); however, this step is followed by a clustering procedure which occasionally aggregates together sequences that are much more divergent than the set threshold. BBH-based approaches are more likely to aggregate highly divergent sequences into the same ortholog as the number of analyzed genomes increases since the clustering step can yield clusters of poorly connected sequences (note that this issue is even more problematic when fusions and fissions of genes occurred and when there is no imposed threshold on sequence length conservation). In contrast, our procedure imposes that every sequence of the orthologous family must be higher than the set threshold (95% identity in this case) relative to the sequence of the pivot genome. This results in a core genome with a narrower sequence identity (not lower than 90% in this case). In fact, we also compared the core genome built by *Roary* with a 95% sequence identity threshold to the core genome obtained by *CoreCruncher* using a 90% sequence identity threshold. This resulted in a larger core genome shared by both methods: 2,640 genes (previously 2,541 when the thresholds were set at 95% sequence identity for both tools). Overall, these results show that *CoreCruncher* is more conservative than BBH-based approaches and that lower sequence thresholds than those typically used for BBH-based methods can be applied.

**Fig. 4.**
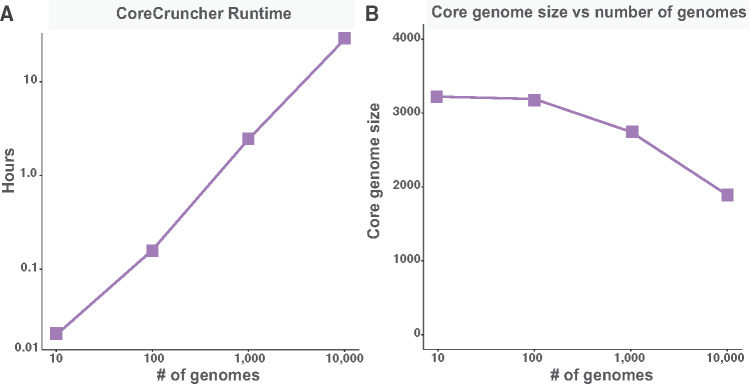
Performance of *CoreCruncher* relative to the size of the data set. The core genomes of different sets of genomes of *E. coli* were built using identical parameters and the same pivot genome (minimum protein identity threshold of 95% and a minimum genome frequency of 90%, nonstringent option). The core genomes were built for four data sets composed of 10, 100, 1,000, and 10,000 genomes of *E. coli* randomly sampled using the same desk computer (Mac Pro) for each *CoreCruncher* run. (*A*) Runtime of the core genomes across the four data sets (note that the *x*-axis and the *y*-axis are both in log-scale). (*B*) Size of the core genome obtained for the four data sets.

Finally, the performance of *CoreCruncher* was assessed by building the core genome of data sets with different sizes. The genomes (protein sequences) of *Escherichia coli* were downloaded from *RefSeq* and used to test the performance of *CoreCruncher* on groups of 10, 100, 1,000, and 10,000 randomly selected genomes using the same parameters, the same pivot genome, and the same desktop computer. We found that the computation time increased approximatively linearly with the size of the genomic data set (fig. 4*A*). Importantly, *CoreCruncher* was able to build the core genome of 10,000 genomes in less than 29 h on a desk computer and yielded a core genome of 1,890 genes. As expected, the size of the core genome decreased with the number of genomes (fig. 4*B*). These results show that *CoreCruncher* is particularly well-suited for the analysis of very large data sets.

## Discussion

*CoreCruncher* is a computational tool that rapidly and robustly assembles core genomes in large genomic data sets and, because it does not conduct all pairwise genome comparisons, *CoreCruncher* is well adapted to current data sets that are becoming increasingly larger (e.g., over 10,000 genomes). Though *CoreCruncher* does not define orthologs by conducting all pairwise genome comparisons, it does not overpredict core genes. On the contrary, the “double outlier” test implemented in *CoreCruncher* yields a more stringent core genome than typical approaches based on BBH. Moreover, *CoreCruncher* can distinguish true orthologs from orthologs containing hidden paralogs or xenologs, a step that is not typically implemented in other tools. For these reasons, *CoreCruncher* constitutes an efficient tool particularly well-suited to analyze current prokaryotic genome data sets composed of hundreds or thousands of genomes.

Importantly, *CoreCruncher* is very fast. Using a desktop computer, the core genome of 484 genomes of *S. marcescens* was built in 2 h 39 min on average, with the longest run being completed in 3 h 43 min. Conversely, over 24 h were needed to analyze each data set with *Roary* using the same computer. It must be noted, however, that these performances cannot be directly compared as *Roary* also builds the entire pan-genome, whereas *CoreCruncher* focuses specifically on building the core genome. Nevertheless, the most time-consuming step of *Roary* consists of conducting the pairwise comparisons (BBH) across genomes; a step that is used by most programs aiming to define orthologs. As *CoreCruncher* does not compute all pairwise comparisons, its running time scales approximately linearly with the size of the data set while, for most programs relying on pairwise comparisons; the running time scales approximately quadratically with the size of the data set (Page et al. 2015). *CoreCruncher* was also tested on very large data sets of 10,000 complete genomes of *E. coli* (using protein sequences) and the program was found to complete core genome construction in under 29 h on a desk computer.

It should be noted that *CoreCruncher* yields more conservative core genomes and our comparison with a BBH procedure showed that the core genes inferred by *CoreCruncher* present a much narrower range of sequence identity. Although this feature is desirable to avoid the inclusion of paralogs and xenologs in the core genome, it might tend to exclude some true core genes from the core genome when run with a strict identity threshold (e.g., 95%). For this reason, we recommend using *CoreCruncher* with more permissive sequence thresholds than those typically used for BBH-based inference (e.g., 90%). We showed that BBH-based methods can infer core genes with highly divergent sequences and these genes are unlikely true core genes. This issue is more prone to occur when building core genomes on large data sets with BBH-based methods due to the clustering step that follows the inference of pairs of orthologs. As a result, we believe that BBH-based methodologies are not best suited to build the core genome of large sets of genomes (i.e., hundreds of genomes or more).

The use of a pivot genome reduces substantially the runtime of the program. It should be noted, however, that using a low-quality genome assembly as a pivot genome can negatively affect the quality of the inferred core genome. Therefore, we strongly encourage the use of a high-quality assembly as the pivot genome. We have also implemented a script (*consensus.py*) that allows users to generate a consensus core genome from two core genomes built with different pivot genomes (note that all other parameters must be strictly identical between the two runs). This procedure prevents the user from missing the detection of core genes that are absent in one of the two pivot genomes. The script also checks the core genomes and excludes potential core genes that were inconsistently inferred by the two runs. Running *CoreCruncher* twice using two pivot genomes and subsequently building the consensus core genome is particularly recommended for data sets containing poor-quality assemblies such as metagenome assembled genomes.

## Materials and Methods

*CoreCruncher* uses *Usearch* (Edgar 2010) (default) or *Blast* (Altschul et al. 1997) to identify homologs based on sequence identity and sequence length by comparing each genome of the data set against the pivot genome. The pivot genome is chosen randomly if not specified. Each gene sequence of the pivot genome is compared against each genome of the data set, and each best hit is considered as a putative ortholog whereas other hits are directly classified as paralogs/xenologs (i.e., within-paralogs/xenologs). For each gene of the pivot genome, all best hits found across the genomes of the data set constitute a putative ortholog and, as such, are associated together in an orthologous family with a single or zero copy per genome. The orthologous family is considered a putative core gene when found in all or nearly all genomes (90% of the genomes by default). This step ultimately results in a putative core genome where no within-paralogs/xenologs are present; however; paralogs and xenologs may still be included due to more complex patterns of gene gains and losses or incomplete genome assemblies—resulting in seeming orthologs (i.e., “hidden paralogs/xenologs”). These cases are expected to occur when the orthologous sequence is lost, but a paralog/xenolog remains present in the genome (fig. 1). Current methodologies based on BBH are unlikely to recognize these sequences as paralogs or xenologs and may include them in the core genome (Kristensen et al. 2011).

First, *CoreCruncher* identifies partially hidden paralogs/xenologs as illustrated in figure 1*C*. Paralogous or xenologous genes can be hidden paralogs/xenologs in some genomes (in instances where the orthologous sequence is absent from the genome) and within-paralogs/xenologs in other genomes. These cases are relatively straightforward to identify: For each putative core gene, the distribution of identity scores of all sequences is built and compared with the identity scores of the within-paralogs/xenologs, that is, each sequence is considered to be an ortholog unless a within-paralog/xenologs with a higher identity score has been identified. In the case where an ortholog presents one or more sequences with a lower identity score than a within-paralog/xenolog, the low-identity sequences are excluded from the orthologous family—which will still be considered a putative core gene if it meets the frequency criterion (i.e., by default an orthologous family must be present in 90% of the genomes to be considered a putative core gene). When *CoreCruncher* is run with the *stringent* option, an orthologous family is automatically excluded from the core genome if a sequence with a lower identity score than a within-paralog/xenolog is detected. Note that this step is only conducted when within-paralogs/xenologs have been identified for a given orthologous gene family.

Second, *CoreCruncher* identifies completely hidden paralogs/xenologs as represented in figure 1*D*. Completely hidden paralogs/xenologs are hidden paralogs or xenologs in one or more genomes (orthologous sequence is absent from the genome(s)) without any within-paralogs/xenologs present in other genomes. To ensure that no hidden paralogs/xenologs are included in the core genome, our method identifies sequences that are significantly more divergent from the other sequences of the orthologous gene, while accounting for the overall divergence of each genome. A given gene sequence may present a higher divergence rate relative to other sequences of the orthologous gene, but this may simply be due to the fact that this gene sequence is present in a more divergent strain. To account for this, we exclude sequences, or an orthologous family, from the core genome if it is itself, or if it contains, a “double outlier,” which is defined in this study as a sequence that is substantially more divergent from 1) the other sequences of the orthologous gene family (fig. 2, distribution 1) and 2) more divergent than the other putative orthologs of the genome (fig. 2, distribution 2). The set of putative core gene sequences is used to draw the distributions of identity scores for each genome that is compared with the pivot genome (distribution 2). The median value of each distribution is used to estimate the overall divergence between each genome and the pivot genome. Then, for each sequence of each putative core gene, we test for the presence of “double outliers,” which, as defined above, is a sequence that is significantly divergent 1) *vertically*: from the other sequences of the orthologous gene (using distribution 1) and 2) *horizontally*: from the average identity score computed across all the putative orthologs relative to the pivot genome (using distribution 2). In both cases, a sequence is defined as an outlier with Tukey’s fences (Tukey 1977): if its identity threshold is below Q1 − 1.5(Q3–Q1) or above Q3 + 1.5(Q3–Q1), with Q1 and Q3 the values of the first and third quartiles, respectively. When a given sequence is inferred as a double outlier, it is considered a hidden paralog/xenolog and this genome’s sequence is excluded from the putative core gene. Other sequences of the putative core gene will still be considered part of the final core genome if they meet the frequency criterion (i.e., by default an ortholog must be present in 90% of the genomes to be considered a core gene). When *CoreCruncher* is run with the *stringent* option, a putative core gene is automatically excluded from the core genome if it contains one or more sequences inferred as a “double outlier.” After filtering out paralogous sequences and/or putative core genes with the “double outlier” test, the final core genome is built.

*CoreCruncher* is implemented with Python 3.7 and is also compatible with Python 2.7 and can run on Mac and Linux operating systems. *CoreCruncher* requires the Python library Numpy and *Usearch* (default) or *Blast* to identify homologs. If specified, the core genes extracted by *CoreCruncher* can be aligned with *muscle* (Edgar 2004) (default) or *mafft* (Katoh and Standley 2013) and these aligned protein or nucleotide sequences will be concatenated into a single merged alignment. *CoreCruncher* is capable of processing either protein or nucleotide sequences and has been found to be robustly capable of building the core genome for large data sets composed of more than 10,000 genomes in less than 29 h on a desk computer (using protein sequences).

## Supplementary Material

Supplementary data are available at *Molecular Biology and Evolution* online.

## Supplementary Material

msaa224_Supplementary_Data
