# Supplementary material for: CoreCruncher: Fast and Robust Construction of Core Genomes in Large Prokaryotic Data Sets
Source: Mol Biol Evol. 2020 Sep 4;38(2):727–34. doi: 10.1093/molbev/msaa224 (PMC7826169; doi:10.1093/molbev/msaa224)
Supplement: msaa224_Supplementary_Data [file msaa224_supplementary_data.zip › msaa224-suppl_data/FigureS2.pdf]

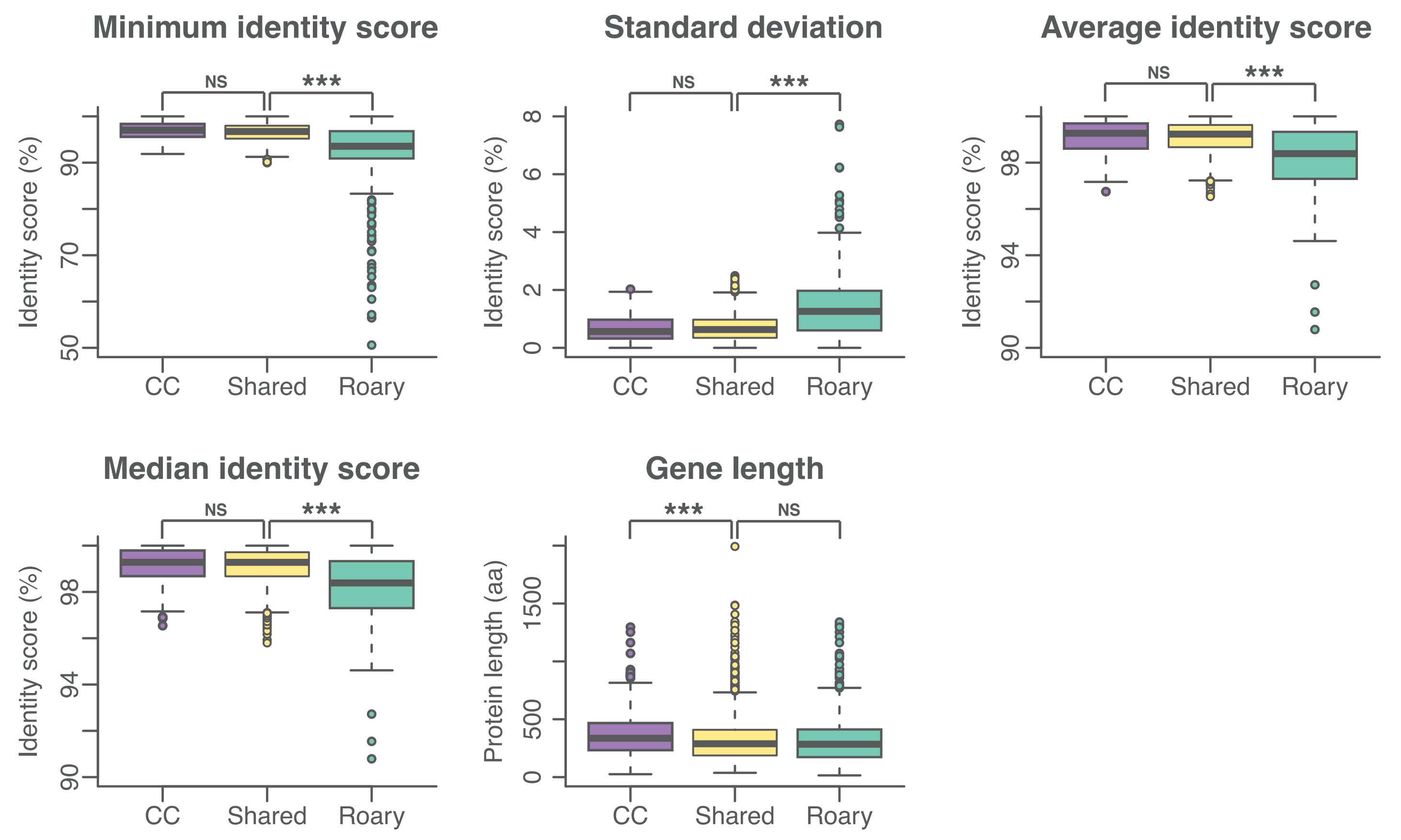
Minimum identity score
NS
***
Identity score (%)
50
70
90
CC
Shared
Roary
Standard deviation
NS
***
Identity score (%)
0
2
4
6
8
CC
Shared
Roary
Average identity score
NS
***
Identity score (%)
90
94
98
CC
Shared
Roary
Median identity score
NS
***
Identity score (%)
90
94
98
CC
Shared
Roary
Gene length
***
NS
Protein length (aa)
0
500
1500
CC
Shared
Roary
