# Supplementary figures and images for: CoreCruncher: Fast and Robust Construction of Core Genomes in Large Prokaryotic Data Sets
Source: Mol Biol Evol. 2020 Sep 4;38(2):727–34. doi: 10.1093/molbev/msaa224 (PMC7826169; doi:10.1093/molbev/msaa224)

### 70% Identity Score

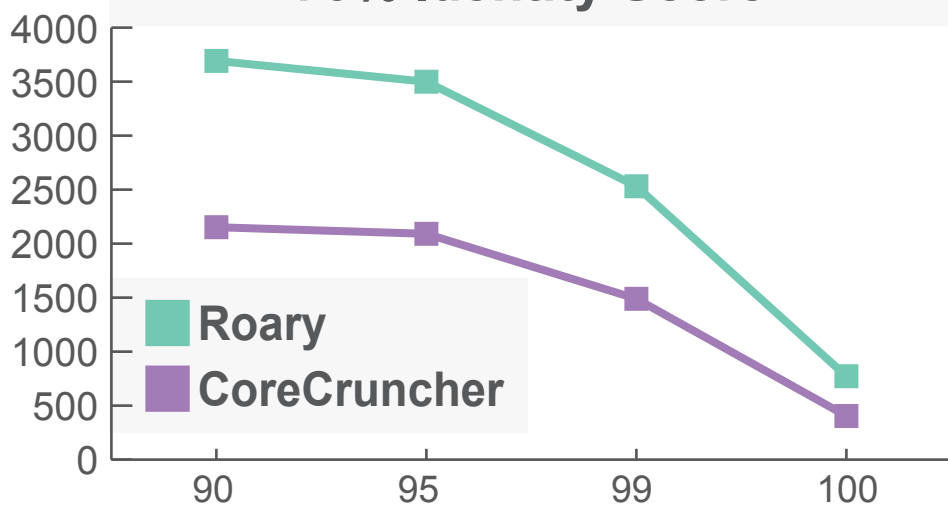

### 90% Identity Score

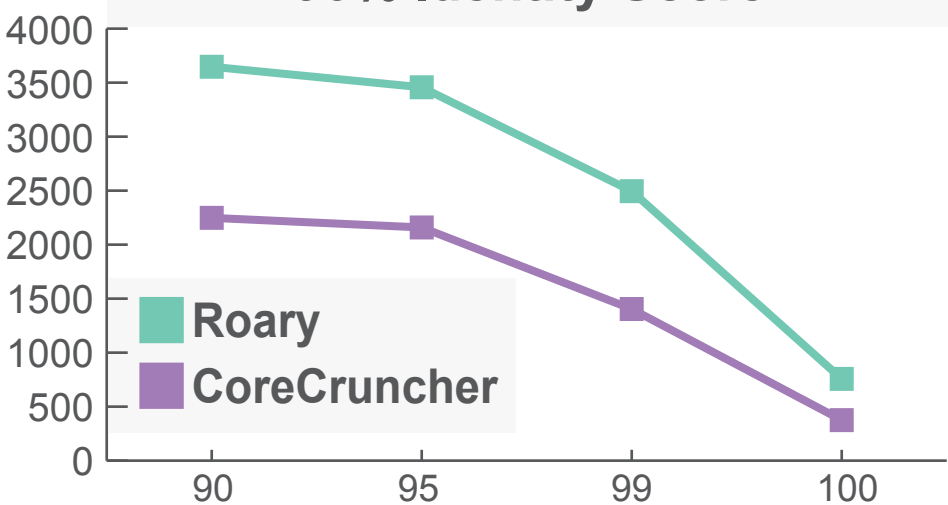

### 95% Identity Score

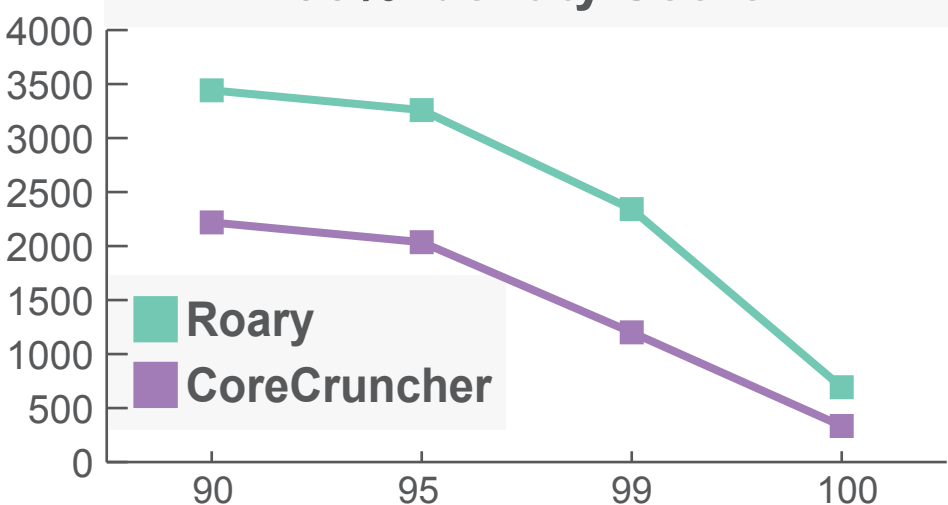

Number of core genes

Frequency

Supplement: msaa224_Supplementary_Data [file msaa224_supplementary_data.zip › msaa224-suppl_data/FigureS1.pdf]
